# Sulphated penta-galloyl glucopyranoside (SPGG) is glycosaminoglycan mimetic allosteric inhibitor of cathepsin G

**DOI:** 10.1093/rpsppr/rqad001

**Published:** 2023-01-06

**Authors:** Rami A. Al-Horani, Daniel K Afosah, Srabani Kar, Kholoud F. Aliter, Madhusoodanan Mottamal

**Affiliations:** 1 Division of Basic Pharmaceutical Sciences, College of Pharmacy, Xavier University of Louisiana, New Orleans, LA, USA; 2 Department of Medicinal Chemistry, School of Pharmacy, Virginia Commonwealth University, Richmond, VA, USA; 3 Department of Chemistry, School of STEM, Dillard University, New Orleans, LA, USA; 4 Department of Chemistry, Xavier University of Louisiana, New Orleans, LA, USA

**Keywords:** cathepsin G, anti-inflammation, allosteric inhibitor, SPGG

## Abstract

**Objective:**

Cathepsin G (CatG) is a cationic serine protease with wide substrate specificity. CatG is reported to play a role in several inflammatory pathologies. Thus, we aimed at identifying a potent and allosteric inhibitor of CatG to be used as a platform in further drug development opportunities.

**Methods:**

Chromogenic substrate hydrolysis assays were used to evaluate the inhibition potency and selectivity of SPGG towards CatG. Salt-dependent studies, Michaelis–Menten kinetics and SDS-PAGE were exploited to decipher the mechanism of CatG inhibition by SPGG. Molecular modelling was also used to identify a plausible binding site.

**Key findings:**

SPGG displayed an inhibition potency of 57 nM against CatG, which was substantially selective over other proteases. SPGG protected fibronectin and laminin against CatG-mediated degradation. SPGG reduced V_MAX_ of CatG hydrolysis of a chromogenic substrate without affecting K_M_, suggesting an allosteric mechanism. Resolution of energy contributions indicated that non-ionic interactions contribute ~91% of binding energy, suggesting a substantial possibility of specific recognition. Molecular modelling indicated that SPGG plausibly binds to an anion-binding sequence of ^109^SRRVRRNRN^117^.

**Conclusion:**

We present the discovery of SPGG as the first small molecule, potent, allosteric glycosaminoglycan mimetic inhibitor of CatG. SPGG is expected to open a major route to clinically relevant allosteric CatG anti-inflammatory agents.

## Introduction

Human cathepsin G (CatG) belongs to a family of cationic serine proteases, which was first identified in the azurophilic granules of neutrophil leukocytes. CatG has a dual trypsin-as well as chymotrypsin-like specificity with preference to Lys, Phe, Arg or Leu as P1 substrate residue. Human CatG is biosynthesised in the form of a 255-amino acid inactive precursor, which contains a signal peptide, an activation peptide at the *N*-terminus, and a *C*-terminal extension. The catalytic activity of CatG depends on a catalytic triad of Ser195, Asp102 and His57 (chymotrypsin numbering).^[1–3]^ CatG is a degradative enzyme that acts intracellularly to digest pathogens and extracellularly to breakdown extracellular matrix components at inflammation sites. CatG has also been reported to activate receptors, platelets, and angiotensin I, amongst others.^[1, 4, 5]^ Importantly, the physiological activity of CatG is regulated by α_2_-macroglobulin, serpin B1, α_1_-antichymotrypsin, α_1_-protienase inhibitor, proteinase inhibitor 6, and secretory leukocyte protease inhibitor.^[3, 6–8]^ Given the wide substrate specificity of CatG, it has been reported to contribute to many diseases such as periodontitis, rheumatoid arthritis, ischaemic reperfusion injury, coronary artery disease and bone metastasis. It is also implicated in acute respiratory distress syndrome, chronic obstructive pulmonary disease, cystic fibrosis and even pain.^[3, 9–11]^

Despite the promise of CatG inhibition in treating and/or managing many diseases, very few inhibitors have been developed including small molecules, peptides, aptamers and sulphated saccharides.^[1]^ In particular, small molecule CatG inhibitors include organophosphorus derivatives, boswellic acid derivatives, 2-substituted saccharines, thiadiazolidinone dioxides^[1]^ and *N*-arylacyl O-sulfonated aminoglycosides,^[12]^ whilst sulphated saccharides include heparin and its derivatives.^[2, 13]^ Mechanistically, organophosphorus derivatives, boswellic acid derivatives, 2-substituted saccharines and thiadiazolidinone dioxides appear to be active site inhibitors, however, *N*-arylacyl O-sulphonated aminoglycosides were reported to be partial mixed inhibitors of CatG with *IC*_*50*_ values of 0.42–209 μM. Importantly, heparin, an anticoagulant sulphated glycosaminoglycan, was characterizsd as allosteric inhibitor with an estimated *K_i_* of <25 pM.^[2, 13]^ Although the potency of heparin inhibiting CatG is very remarkable, its anti-CatG-mediated anti-inflammatory activity is of limited clinical utility given the high risk of excessive bleeding. Therefore, we hypothesised that a sulphated penta-galloyl glucopyranoside (SPGG), a small molecule mimetic of heparin, may serve as a potent and allosteric inhibitor of CatG with no significant risk of bleeding, given its unique bleeding free-anticoagulant mechanism of factor XIa (FXIa) inhibition.^[14, 15]^

Accordingly, we investigated SPGG’s potential of inhibiting CatG and found that it inhibited CatG in a salt-dependent fashion with an *IC*_*50*_ value of 57 ± 5 nM and an efficacy of ~90% under physiological conditions. SPGG demonstrated significant selectivity over other digestive and clotting serine proteases. Its activity was also found to be physiologically relevant as it did protect fibronectin and laminin against CatG-mediated degradation. Similar to heparin and its derivatives, Michaelis–Menten kinetics indicated that SPGG is allosteric inhibitor of CatG. Binding affinity calculations show that CatG inhibition by SPGG is driven by both ionic and non-ionic (H-bond) interactions between sulphate groups of SPGG with their counterparts Arg and/or Lys of CatG. Overall, SPGG serves as a lead molecule for the development of more potent, selective, and allosteric inhibitors of CatG for clinical use in inflammatory pathologies.

## Materials and Methods

### Materials

Human CatG was purchased from Enzo life sciences (Farmingdale, NY). Human plasma clotting enzymes and digestive enzymes were purchased from Haematologic Technologies (Essex Junction, VT). Laminin, fibronectin and the chromogenic substrate for CatG (S-7388) were purchased from Sigma Aldrich (St. Louis, MO). The chromogenic substrates for thrombin, FIIa, FVIIa, FXa, FIXa and FXIIa were purchased from Biomedica Diagnostics (Windsor, NS Canada). FXIa chromogenic substrate (S-2366) and trypsin chromogenic substrate (S-2222) were purchased from Diapharma (West Chester, OH). Pre-cast SDS-PAGE gels were from BioRad (Hercules, CA). Coomassie brilliant blue for gel electrophoresis was from Fisher Scientific (Waltham, MA). Stock solutions of CatG were prepared in 20 mM Tris-HCl buffer, pH 7.4 containing 0.02% Tween80, 0.1% PEG8000, 2.5 mM CaCl_2_ and 100 mM NaCl. All experiments were repeated at least two times.

### Chemical synthesis of SPGG

SPGG was quantitatively synthesized as reported previously.^[14, 16]^ Briefly, SPGG was synthesized using a three-step protocol involving DCC-mediated esterification of *β*-d-glucopyranose with 3,4,5-tribenzyloxybenzoic acid followed by palladium-catalyzed hydrogenation to obtain penta-galloyl glucopyranoside. The latter intermediate was sulphonated under microwave conditions for 2 h at 90°C using trimethylamine–sulphur trioxide complex to prepare SPGG. The product was characterized by ^1^H- and ^13^C-NMR as well as by high resolution mass spectroscopy. Results indicated that the chemical identity of the molecule synthesized in this report is consistent with that of SPGG in previous reports.^[14, 16]^

^1^H-NMR (D_2_O, 400 MHz): 8.11–7.40 (m, 10 H), 6.51–6.47 (m, 1 H), 6.11–6.18 (m, 1 H), 5.79–5.97 (m, 2 H), 4.85–4.60 (m, 3 H). ^13^C-NMR (D_2_O, 100 MHz): 166.39, 165.70, 165.40, 164.71, 150.62, 150.53, 147.82, 147.43, 147.17, 145.69, 145.53, 126.34, 122.42, 122.22, 122.17, 121.98, 120.97, 119.74, 118.99, 118.69, 115.32, 93.04, 74.5, 72.24, 71.59, 68.90, 63.50.

### Inhibition of CatG by SPGG using a chromogenic substrate hydrolysis assay

Direct inhibition of CatG was determined at pH 7.4 and 37°C by a chromogenic substrate hydrolysis assay, as described previously.^[17]^ To each well of a 96-well microplate containing 88 μl of 20 mM tris buffer containing 100 mM NaCl, 2.5 mM CaCl_2_, 0.1% PEG 8000 and 0.05% tween 80 was added 3 μl of CatG (final concentration of 30 nM), and 5 μl of H_2_O or SPGG (final concentration of 0–100 μM). Following a 5-min incubation, 3 μl of CatG substrate was added (final concentration 750 μM) and the residual CatG activity was obtained from the initial rate of increase of absorbance at 405 nm. The relative residual activity of CatG at each of the SPGG concentrations was obtained from the ratio of CatG activity in the presence and absence of SPGG. Logistic Equation (1)^[17]^ was used to plot the dose-dependence curve to obtain the *IC_50_* (potency) and efficacy of CatG inhibition. Here, *Y* is the ratio of residual CatG activity in the presence of SPGG to that in its absence, *Y*_O_ and *Y*_M_ are the minimum and maximum values of fractional residual CatG activity, respectively, IC_50_ is the concentration of SPGG that results in 50% inhibition of CatG activity and HS is the Hill slope.


(1) $$Y=Y_{\text{O}}+\frac{Y_{\text{M}}-Y_{\text{O}}}{1+10^{\left(\log{\left[\text{I}\right]}_{0}-\log{\text{IC}}_{50}\right)\left(\text{HS}\right)}}.$$

### Salt dependence of SPGG inhibition of CatG

The direct inhibition of CatG cleavage of a chromogenic substrate was determined at 37°C, as described above, in pH 7.4 Tris buffer containing 2.5 mM CaCl_2_, 0.1% PEG 8000, 0.05% tween 80 and 50–500 mM NaCl. The *K_i_* (nM) values were estimated using the equation in https://bioinfo-abcc.ncifcrf.gov/IC50_Ki_Converter/index.php.^[18]^ Slope, *Z* and intercept were calculated from linear regressional analysis of log *K*_i,calculated_ versus log[NaCl] as defined by equation: Log *(K_i_* (M)) = Log (*K_i_* (M) (non-ionic)) + *Zψ* Log ([NaCl] (M)); *ψ* = 0.8 [32]. The contributions of ionic and non-ionic binding energies to the interactions were obtained from slope and intercept of the linear plot of log *K*_D’obs_ versus log [Na+], according f to Equation (2). In this equation, *K*_D,NI_ is the dissociation constant at [Na+] = 1 M and slope “*m*” = *Z* × *ψ*, where *Z* is the number of ion-pairs formed upon binding and *ψ* is the fraction of monovalent counterions released per negative charge following interaction.^[19, 20]^

(2) $$\log\;K_{\text{D},\text{obs}}=\log\;K_{D,NI}+m\times \log\;\left[{\text{Na}}^{+}\right].$$

### Inhibition of selected coagulation and digestive serine proteases by SPGG

Direct inhibition of thrombin (FIIa), factor VIIa (FVIIa), factor IXa (FIXa), factor Xa (FXa), FXIa, factor XIIa (FXIIa), trypsin and chymotrypsin using the corresponding chromogenic substrate hydrolysis assays as previously reported.^[14, 17, 21, 22]^ Briefly, to each well of a 96-well microplate containing 85–185 μl of pH 7.4°C Tris buffer containing 100 mM NaCl, 2.5 mM CaCl_2_, 0.1% PEG 8000, and 0.05% tween 80 at 25 (thrombin) or 37°C (FVIIa, FIXa, FXa, FXIa, FXIIa, chymotrypsin, and trypsin) were added 5 μl of enzyme (thrombin, FVIIa, FIXa, FXa, FXIa, FXIIa, chymotrypsin, and trypsin) and 5 μl of SPGG. Following an incubation period of 5 min, the appropriate chromogenic substrate (Spectrozyme TH, Spectrozyme FIXa, Spectrozyme FXa, S-2366, Spectrozyme FXIIa and S-2222) were added and the residual enzyme activity was measured from the initial rate of increase in absorbance at 405 nm. Relative residual enzyme activity as a function of SPGG concentration was fitted using Logistic Equation (1) to obtain the *IC_50_* (potency), Δ*Y*% (efficacy) of enzyme inhibition, and HS (Hill slope). The concentrations of enzymes and substrates in microplate cells were: 6 nM and 50 μM for thrombin; 1.09 nM and 125 μM for FXa; 5 nM and 125 μM for FXIIa; 89 nM and 850 μM for FIXa; 8 nM and 1000 μM for FVIIa (along with 40 nM recombinant tissue factor); 72.5 ng/ml and 80 μM for bovine trypsin; and 500 ng/ml and 240 μM for bovine chymotrypsin. See Supplementary information for tabulated concentrations and molecular formula of the chromogenic substrates.

### Michaelis–Menten enzyme kinetics

The initial rate of the hydrolysis of the chromogenic substrate by CatG was monitored from the linear increase in absorbance corresponding to less than 10% consumption of substrate at 37°C in pH 7.4 20 mM Tris buffer containing 100 mM NaCl, 2.5 mM CaCl_2_, 0.1% PEG 8000 and 0.05% tween 80. The initial rate was measured at various substrate concentrations (0–2500 mM) at fixed enzyme concentration (30 nM), and fixed SPGG concentrations (0, 25, 50, 75, 100 and 200 nM). The data were fitted by the Michaelis–Menten Equation (3) to determine the *K*_M_ (substrate affinity) and *V*_MAX_ (maximum reaction velocity).


(3) $$V=\frac{V_{\text{MAX}}\left[S\right]}{K_{\text{M}}+\left[S\right]}.$$

### Inhibition of CatG cleavage of laminin and fibronectin by SPGG

Inhibition of CatG cleavage of laminin and fibronectin by SPGG was studied using SDS-PAGE, as previously reported.^[23, 24]^ Briefly, CatG (0.8 or 0.5 μM) was incubated with different concentrations of SPGG (final concentrations; 0 μM, 10 μM, 100 μM and 1000 μM), and laminin (20 μg) or fibronectin (36 nM). Following incubation for 60 min, the samples were quenched using SDS-PAGE loading buffer containing DTT and subjected to electrophoresis on 10% SDS-PAGE pre-cast gels. The gels were visualized by silver staining.

### Molecular modelling studies

Structure-based molecular docking studies were conducted to identify the binding mode of SPGG to CatG. The Molecular Operating Environment (MOE) 2020 software suite was used for all the docking experiments.^[25]^ The protein structure was obtained from the crystal structure of human cathepsin G in complex with a peptidyl phosphonate inhibitor (PDB ID: 1CGH).^[26]^ The initial protein structure for docking was prepared by removing the crystal water molecules followed by 3D protonation at a physiologic pH of 7 and energy minimization. The minimized protein structure was used as the receptor for docking studies. The 3D structure of the SPGG molecule was prepared using the builder module of MOE, and subsequently, its energy was minimized to an RMS gradient of 0.1 kcal/mol using the Amber 10:EHT force filed. Considering the structural flexibility of the SPGG molecule, a conformational analysis was conducted at the default setting using the Low Mode MD method implemented in MOE and an RMS gradient of 0.005. All generated conformations were subjected to docking experiments. Using Cardin and Weintraub’s determination of heparin-binding sequences,^[27]^ the consensus amino acid sequence having the XBBXBX motif, where B is a basic residue and X is hydropathic residue, was selected as the purported binding site for SPGG, which is ^112^VRRNRN^117^. But having found two Arg ahead of V^112^, this motif was extended and selected ^109^SRRVRRNRN^117^ sequence to define the binding site. The default parameters were used to do the docking experiments. The best binding pose based on the docking score was selected to study the molecular level interactions of SPGG molecule with CatG.

## Results and Discussion

### Rationale for the current study and the chemical synthesis of SPGG

**Figure 1.**
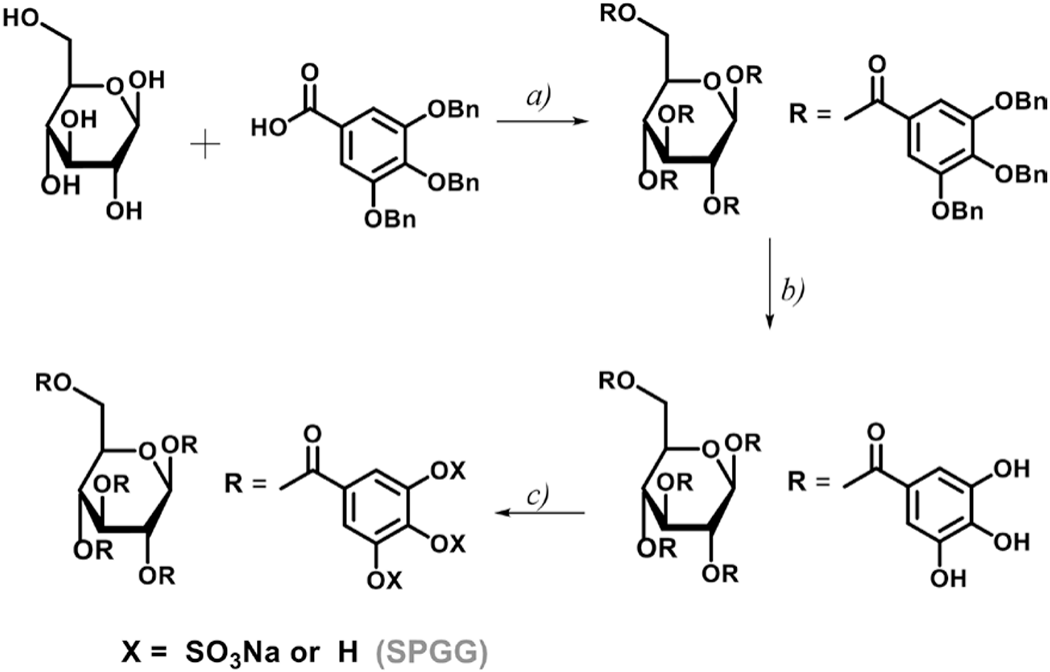
Chemical synthesis of SPGG. (a) 3,4,5-Tribenzyloxybenzoic acid (5 equiv), DCC (5 equiv), DMAP (5 equiv), CH_2_Cl_2_, reflux, 24 h, 85–90%; (b) H_2_ (g) (50 psi), Pd(OH)2/C (20%), CH_3_OH/THF, rt, 10 h, >92%; (c) N(CH_3_)_3_-SO_3_ (5 equiv/OH), CH_3_CN (2 ml), MW, 90°C, 2 h, 66–72%.

The fundamental idea in discovering small molecule allosteric CatG inhibitors was to screen glycosaminoglycan mimetics that potentially bind in a heparin-like fashion and induce an inhibitory conformational change in the active site. Heparin, was characterized as a tight-binding, allosteric inhibitor with an estimated *K_i_* of < 25 pM.^[2, 13]^ In this context, ~20% of the heparin–CatG binding energy was due to ionic interactions, and an average of two ionic interactions was required for a 1:1 heparin–CatG complex.^[28]^ Despite the inhibitory activity of heparin and its derivatives, their use as anti-inflammatory drugs has serious limitations due to their strong anticoagulant properties that come with significant risk of excessive bleeding and other side effects.^[29]^ To overcome these limitations, we have previously developed a number of small molecule heparin mimetics that are associated with little-to-none bleeding including SPGG.^[14–16]^ The molecule demonstrated bleeding free-anticoagulant activity by targeting human FXIa. The molecule also demonstrated promising antiviral and antimicrobial activities.[^30, 31]^ Considering the size of the allosteric anion-binding sites on other heparin-binding proteins such as thrombin and FXIa, we reasoned that the potential allosteric anion-binding site on CatG has a similar size, and thus, we studied the potential inhibition of CatG by SPGG, which was synthesized as reported earlier (Figure 1).^[14–16]^

In particular, penta-galloyl glucopyranoside was sulphated in CH_3_CN using trimethylamine–sulphur trioxide complex. The reaction mixture was microwaved at 100°C for 2 h. It was then purified using size exclusion chromatography and the sodium salt form was generated by sodium exchange chromatography. SPGG was characterized by ^1^H- and ^13^C-NMR as well as mass spectroscopy and chemical characteristics were identical to those reported previously.^[14–16]^ SPGG was found to be predominantly decasulfated with an average molecular weight of 2178.

### Direct inhibition of CatG by SPGG and its selectivity over other serine proteases

**Figure 2.**
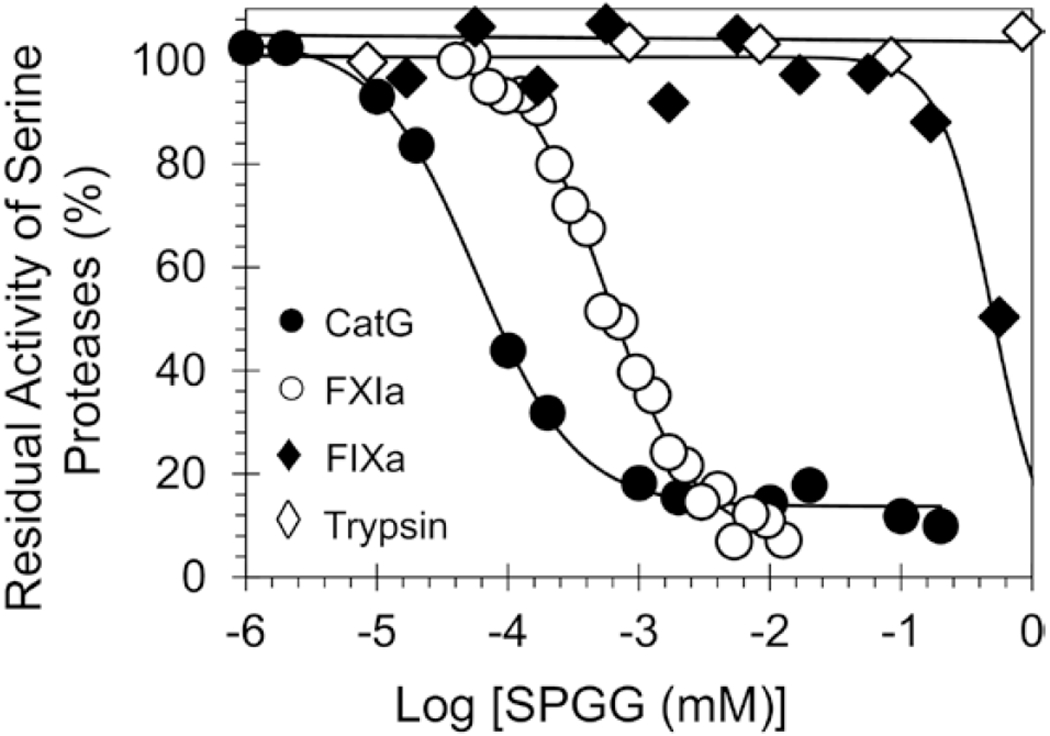
Direct inhibition of human CatG (and other serine proteases) by SPGG. The inhibition profiles of (●) CatG, (○) FXIa, (♦) FIXa and (◊) trypsin were studied using the corresponding chromogenic substrate hydrolysis assays as described in the experimental part. Solid lines represent sigmoidal dose–response fits (Equation (1)) of the data to obtain the values of *IC_50_*, HS and ΔY.

SPGG was evaluated for its potential to inhibit CatG hydrolysis of S-7388, a chromogenic small peptide substrate, at 37°C and pH 7.4, as reported in our previous studies.^[17]^ The presence of SPGG resulted in a dose-dependent reduction in CatG activity (Figure 2). The dose-dependence inhibition of CatG activity could be fitted using the logistic Equation (1), which resulted in an *IC_50_* of 57 ± 5 nM with an efficacy of ~90% and Hill slope of 1.1 (Table 1), at salt concentration of 100 mM.

To establish the selectivity profile of SPGG, its inhibition potential towards other serine proteases was established using the corresponding chromogenic substrate hydrolysis assays for digestive enzymes (chymotrypsin and trypsin) and clotting factors (factors XIIa, XIa, Xa, IXa, VIIa and IIa), as described in our previous studies.^[14, 17, 21, 22]^ The inhibition potential in all assays was determined by spectrophotometric analysis of the residual serine protease activity in the presence of different concentrations of SPGG. In addition to CatG, Figure 2 displays the decrease in FXIa, FIXa and trypsin activity over a wide range of SPGG, which was fitted using Equation (1) to calculate the corresponding *IC*_50_, if any (Table 1). SPGG was found to be a weaker inhibitor for FXIIa, FXIa, FXa and FIXa. Particularly, SPGG demonstrated selectivity index (SPGG-IC_50_ CatG/SPGG-IC_50_ protease) of 2053-fold over FXIIa, 10-fold over FXIa, 2146-fold over FXa and 8818-fold over FIXa. In contrast, SPGG did not inhibit chymotrypsin, trypsin, FVIIa or thrombin at the highest concentrations tested. These results suggested that SPGG is a selective inhibitor for CatG.

### CatG inhibition by SPGG is physiologically relevant. Effect of SPGG on CatG-mediated proteolysis of laminin and fibronectin

**Figure 3.**
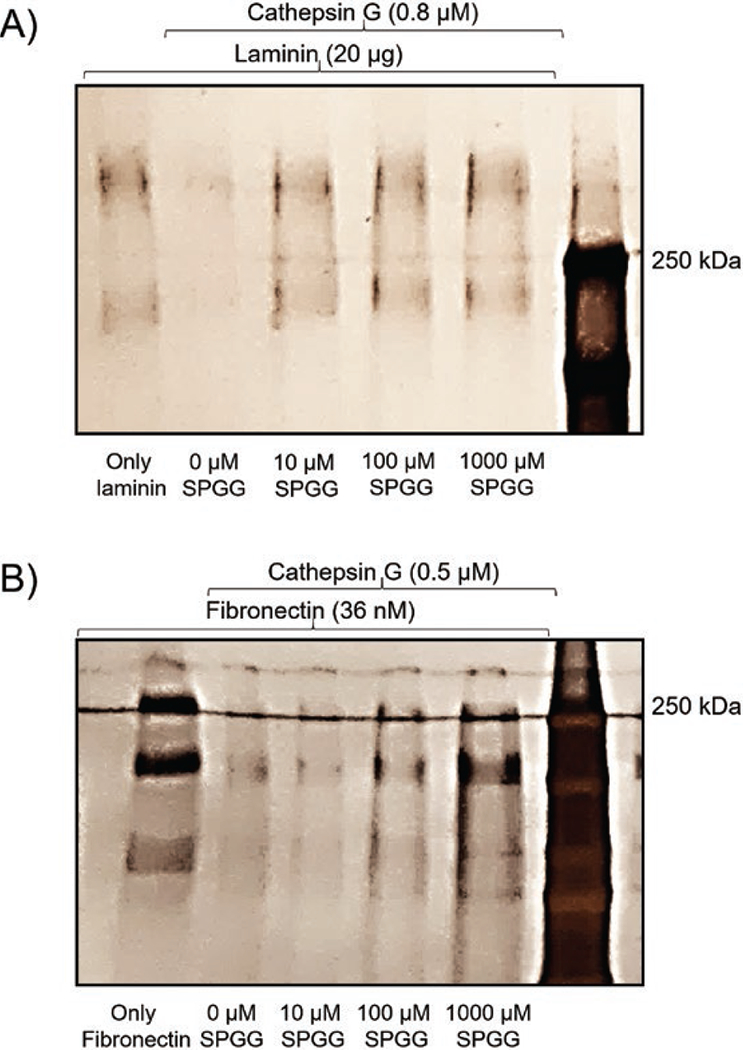
(A) Effect of SPGG on CatG-mediated hydrolysis of laminin as evaluated by SDS-PAGE under reducing conditions. SPGG concentrations used were 0, 10, 100 and 1000 μM. (B) Effect of SPGG on CatG-mediated hydrolysis of fibronectin as evaluated by SDS-PAGE under reducing conditions. SPGG concentrations used were 0, 10, 100 and 1000 μM.

Although SPGG inhibits CatG hydrolysis of chromogenic substrate, extracellular matrix (ECM) components serve as more relevant substrates of CatG. In fact, based on their *in vitro* properties, we reasoned that SPGG could protect extracellular matrix components from proteolysis mediated by proteinases activated during the inflammatory process including CatG. Accordingly, we studied the *in vitro* effect of SPGG on the CatG-mediated proteolysis of laminin and fibronectin, the major non-collagenous components of ECM and basement membranes.^[32]^ SDS-PAGE analyses showed that laminin was significantly cleaved by CatG, as evidenced by the disappearance of the ≥200-kDa laminin bands (Figure 3a, lane 2). In the presence of SPGG (10–1000 μM), however, laminin is protected completely from cleavage by CatG (Figure 3a, lanes 3–5). Likewise, SDS-PAGE analyses showed that fibronectin was significantly cleaved by CatG, as evidenced by the disappearance of fibronectin bands (Figure 3b, lane 2). In the presence of SPGG (100–1000 μM), fibronectin is protected from CatG-mediated cleavage (Figure 3a, lanes 4–5). Together, SDS-PAGE analyses indicate that SPGG inhibition of CatG is physiologically relevant.

### SPGG is an allosteric inhibitor of CatG

**Figure 4.**
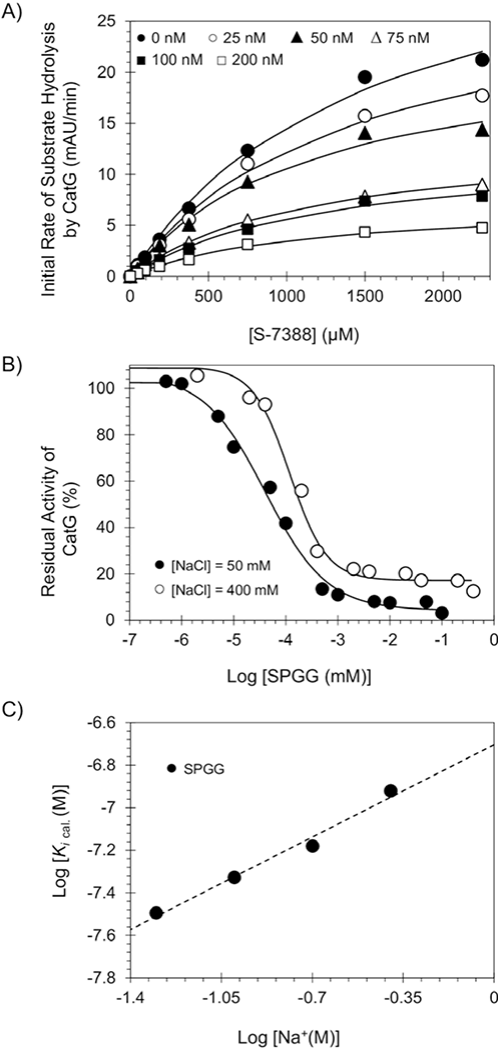
(A) Michaelis–Menten kinetics of S-7388 hydrolysis by human CatG in the presence of SPGG. The initial rate of hydrolysis at various substrate concentrations was measured in pH 7.4 buffer as described in the experimental part using the wild-type full-length human CatG. SPGG concentrations are 0 (●), 25 (○), 50 (▲), 75 (Δ), 100 (■) and 200 nM (□). Solid lines represent non-linear regressional fits to the data using the standard Michaelis–Menten equation to calculate the *V*_MAX_ and *K*_M_. (B) Salt-dependent direct inhibition of CatG by SPGG, using the corresponding chromogenic substrate (S-7388) hydrolysis assay. Salt concentrations used are 50 mM (●) and 400 mM (○). Solid lines represent sigmoidal fits to the data to obtain the inhibition values using Equation (1). (C) Dependence of the calculated equilibrium dissociation constant of SPGG–CatG complex on the concentration of sodium ion in the medium at pH 7.4 and 37°C. The *K*_D,obs_ of SPGG binding to human CatG was calculated. Solid lines represent linear regression fits using Equation (3). Error bars in symbols represent standard deviation of the mean from at least two experiments. Symbols without apparent error bars indicate that the standard error was smaller than the size of the symbol.

To understand the mechanistic basis of inhibition, Michaelis–Menten kinetics of S-7388 hydrolysis by CatG was performed in the presence of SPGG at pH 7.4 and 37°C. Figure 4a shows the initial rate profiles in the presence of SPGG (0–200 nM). Each curve shows a characteristic rectangular hyperbolic dependence, which could be fitted using Michaelis–Menten equation (Equation (3)) to obtain the apparent *K*_M_ and *V_MAX_* (Table 2). The *K_M_* for S-7388 remained essentially unchanged in the presence or absence of SPGG, whilst the *V_MAX_* decreased steadily from 37.2 ± 3.2 mAU/min in the absence of SPGG to 7.3 ± 0.4 mAU/min at 200 nM of SPGG (~5.5-fold decrease). Therefore, SPGG appears to bring about structural changes in the active site of CatG, which does not affect the formation of Michaelis complex, but induces a significant dysfunction in the catalytic apparatus. This indicates that SPGG is an allosteric inhibitor of CatG.

### Salt-dependent inhibition of CatG by SPGG

Although the SPGG–CatG interaction is likely to be electrostatically driven, non-ionic forces may contribute to a significant extent, as noted for heparin–CatG,^[28]^ SPGG–FXIa interaction^[33]^ and heparin–antithrombin.^[19]^ A significant non-ionic binding energy component increases the interaction specificity because the majority of non-ionic forces, for example, cation-*π* interactions, H-bonding and others depend strongly on the orientation and the distance of interacting pair of molecules.^[34, 35]^ In contrast, ionic bonds are non-directional and less dependent on distance, which may increase initial interaction but offer less selectivity of recognition. To determine the nature of interactions between SPGG and CatG, the *IC*_50_ values were measured as a function of the ionic strength of the medium at pH 7.4 and 37°C. The *IC_50_* value of SPGG towards CatG was measured spectrophotometrically under various salt concentrations (50, 100, 200 and 400 mM), as described above. Interestingly, a 2-fold decrease in salt concentration led to a ~1.36-fold increase in the potency of SPGG (Figure 4b and Table 3). Likewise, a 2-fold increase in the salt concentration to 200 mM and then to 400 mM led to ~1.33 decrease in potency (*IC_50_* = 76 nM) and then to another ~1.71 drop in the potency of inhibition (*IC*_50_ = 130 nM), respectively (Figure 4b and Table 3). Subsequently, the corresponding *K_i_* (nM) values were estimated using a formula previously reported following a classical model of noncompetitive enzyme inhibition ^[18]^ and the results are reported in Table 4.

The protein–polyelectrolyte theory ^[19, 20]^ indicates that the contribution of non-ionic forces to an interaction, similar to that of CatG–SPGG, can be quantified from the intercept of a double log plot (Figure 4c). The slope of the linear profile corresponds to the number of ion-pair interactions (*Z*) and the fraction of monovalent counterions released per negative charge following ligand binding (*Ψ*), whilst the intercept corresponds to the non-ionic affinity (*K_i(non-ionic)_*). SPGG exhibited a slope of 0.621 and intercept of −6.7034 (Table 4). This indicates a binding energy due to ionic forces (Δ*G_(ionic)_*) of ~0.885 kcal/mol at pH 7.4, and a binding energy due to non-ionic forces of ~9.498 kcal/mol (Δ*G_(non-ionic)_*). The result for SPGG interacting with CatG is similar to that for heparin. Although each of these molecules is highly negatively charged, the resolution of the nature of forces involved in recognition shows that nearly 91.5% of binding energy for SPGG arises from non-ionic forces. The non-ionic contribution is ~80% for heparin. The number of ion-pairs formed in the interaction for SPGG is 0.77625, which is similar to that of heparin.^[2, 13, 28]^ This suggests that SPGG most probably utilizes site(s) on CatG similar to heparins. SPGG is the first small glycosaminoglycan mimetic with such a high non-ionic binding energy contribution and may encompass interactions that afford a highly selective recognition. The origin of the non-ionic interactions is unclear at the present time, nevertheless, the majority of forces most likely arise from H-bonds with multiple sulphate groups. It is less likely that cation-*π* interactions play a substantial role in SPGG interactions because such interactions are non-existent for heparin which also exhibits high proportion of non-ionic interactions.

### Molecular modelling studies

**Figure 5.**
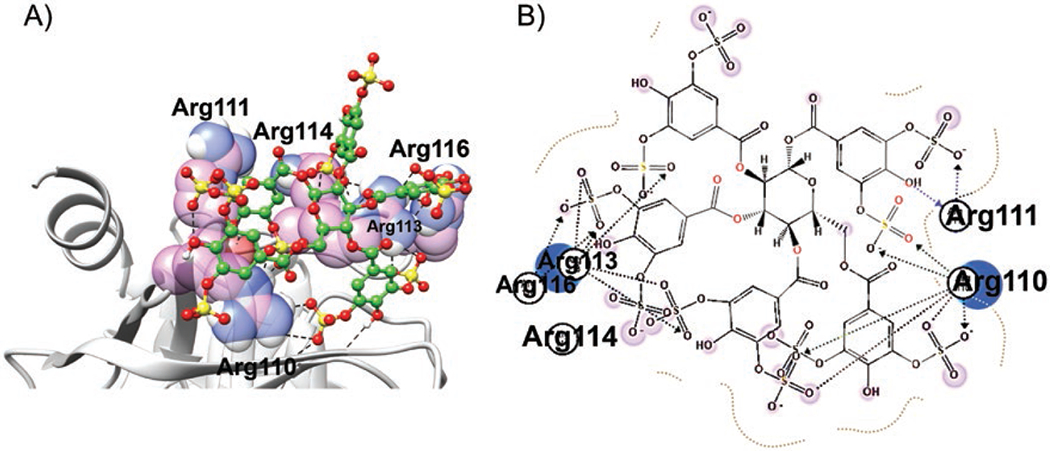
(A) Cartoon representation showing SPGG’s interactions with Arg residues of a potential CatG’s anion-binding site. SPGG is represented as spheres in which carbon atoms are depicted in green spheres, sulphur atoms are depicted in yellow spheres, and oxygen atoms are depicted in red spheres. (B) Two-dimensional representation of SPGG’s interactions with the putative Arg-rich binding site of ^109^SRRVRRNRN^117^.

To identify a plausible binding mode for SPGG on CatG, we performed molecular docking studies, as described in the experimental part, by considering Cardin and Weintraub’s determination of heparin-binding sequences.^[27]^ The consensus amino acid sequence having the XBBXBX (^112^VRRNRN^117^) motif was initially selected as the putative binding site for SPGG. Added to this sequence are two Arg residues that precede V112. Accordingly, ^109^SRRVRRNRN^117^ sequence was eventually selected to perform the molecular modelling studies. As a result, SPGG was found to reasonably fit into this sequence (Figure 5a). In fact, SPGG has shown multiple salt bridge and/or H-bond with several Arg residues. About 7 out of the 10 sulphate groups of SPGG appear to interact with Arg110, Arg111, Arg113 and Arg116 residues. Interestingly, each benzoyl moiety at positions-1 and -4 of the sugar central moiety offers two sulphate groups for interaction with CatG, however, each benzoyl moiety at positions-2, -3 and -6 offers only one of their sulphate groups to interact with CatG (Figure 5b). Although we report no mutagenesis studies or X-ray crystallography results, binding in this region is more likely based on best scores obtained. Conceptually, considering the results of the above molecular modelling studies, we should theoretically be able to design a hepta-sulphated inhibitor that maintains high potency and efficacy. This should further facilitate aspects related to pharmaceutical development.

## Conclusion and Future Directions

CatG is a promising drug target to design inhibitors to treat and/or manage many inflammatory diseases, yet few inhibitors have been developed. One interesting platform to develop CatG inhibitors is glycosaminoglycans and their non-saccharide mimetics. This idea was inspired by heparin, an anticoagulant sulphated glycosaminoglycan, which was characterized as allosteric CatG inhibitor with an estimated *K_i_* of <25 pM.^[2, 13]^ However, the life-threatening bleeding risk of heparin has hindered its development as anti-inflammatory. To overcome this issue, we opted to test SPGG, a small molecule heparin mimetic with bleeding free-anticoagulant and antiviral activities.^[14, 31]^ Accordingly, we investigated SPGG’s potential of inhibiting CatG and found that it inhibited CatG in a salt-dependent fashion with an *IC_50_* of ~57 nM and an efficacy of ~90%. SPGG demonstrated significant selectivity over other serine proteases. Its activity was also found to be physiologically relevant as it did protect fibronectin and laminin against CatG-mediated degradation.

A mechanistic aspect that adds significantly to the clinical viability of SPGG is allostery. Allostery provides a unique prospect of highly selective recognition, which is exploited by nature to an advantage.^[36–38]^ In comparison to orthosteric sites, allosteric sites tend to be less conserved in a family of homologous proteins. For example, the allosteric sites of serine protease clotting factors such as factors IIa, Xa, IXa and XIa display considerable sequence variability,^[39]^ despite possessing a fairly similar trypsin-like, active site specificity. This greatly enables selective targeting of an allosteric site. Michaelis–Menten kinetics revealed a classic allosteric inhibition mechanism (Figure 4a). SPGG’s allostery appears to arise from binding to an anion-binding site as shown by the salt-dependent inhibition studies (Figure 4b). We recruited molecular modelling studies to identify a plausible binding site for SPGG involving the sequence ^109^SRRVRRNRN^117^ (Figure 5). Nevertheless, future work using co-crystallography and/or alanine scanning mutagenesis should help pinpoint the residues involved in interaction with SPGG. Furthermore, a significant advantage of allosteric inhibitors is the prospect of modulation. Given that allostery involves coupling of two sites, that is, the inhibitor’s allosteric binding site and the catalytic site, the nature and the extent of coupling is significantly dependent on the structure of the inhibitor. This suggests that whereas some allosteric modulators may induce ~100% inhibition, others may only be partially efficacious. This is important when designing inhibitors that target enzymes with multiple functions at multiple sites.

Another interesting aspect relevant to SPGG resulted from the analysis of forces contributing to CatG–SPGG interaction that led to a rather unexpected result. Despite the presence of numerous sulphate groups on a small scaffold, ionic forces were not the dominant contributors. This work adds to the growing body of evidence that aromatic mimetics of glycosaminoglycans inherently bind proteins with higher non-ionic binding energy, which is expected to induce higher specificity of interaction.^[33]^ A unique and important advantage of SPGG is that it is readily synthesizable. In this work, SPGG was chemically synthesized in three quantitative chemical steps from d-β-glucopyranoside and 3,4,5-tribenzyloxy-benzoic acid. This raises a strong possibility that SPGG can be obtained on a large scale in a relatively inexpensive manner.

Overall, SPGG is an allosteric inhibitor of CatG that displays good potency and selectivity. It possesses many advantages including relatively easy synthesis, allosteric recognition and high specificity of targeting CatG. SPGG is likely to open up new opportunities for the design of clinically relevant allosteric anti-inflammatory agent. SPGG will be tested in appropriate *in vivo* models of inflammation diseases, and the results will be reported in due time. As mentioned earlier, the diseases that may benefit from CatG inhibitors include rheumatoid arthritis, ischaemic reperfusion injury, coronary artery disease, acute respiratory distress syndrome, chronic obstructive pulmonary disease and cystic fibrosis. Finally, given the anticoagulant, antiviral and anti-inflammatory effects of SPGG, it may be worth testing SPGG in the co-pathologies of inflammation, coagulation and infections such as COVID-19.

## Supplementary Material

Suppl Info

## Figures and Tables

**Table 1:** Inhibition of different serine proteases including CatG by SPGG under physiological conditions.^1^

Enzyme	IC_50_ (μM)	HS	*ΔY* (%)
CatG	0.057 ± 0.005^2^	1.1 ± 0.1	90 ± 2
Chymotrypsin	>840	*ND* ^3^	*ND*
Trypsin	>840	*ND*	*ND*
FXIIa	117.5 ± 26.5	3.0 ± 1.8	56 ± 6
FXIa	0.56 ± 0.03	1.2 ± 0.1	98 ± 3
FXa	122.3 ± 69.9	0.8 ± 0.3	100 ± 2
FIXa	502.6 ± 95.8	2.1 ± 0.9	100 ± 14
FVIIa	>1680	*ND*	*ND*
Thrombin	>500	*ND*	*ND*

1 The inhibition parameters were obtained following non-linear regression analysis of direct inhibition of the serine protease. Enzyme inhibition was evaluated by spectrophotometric analysis of residual enzyme activity.

2 Errors represent ± 1 S.E.

3 Not determined.

**Table 2:** Michaelis–Menten Kinetics of substrate hydrolysis by CatG in the presence of SPGG.^1^

[SPGG] (nM)	*K*_M_ (mM)	*V*_MAX_ (mAU/min)
0	1.54 ± 0.25^2^	37.2 ± 3.2
25	1.43 ± 0.25	29.8 ± 2.6
50	1.14 ± 0.22	22.8 ± 2.0
75	1.15 ± 0.07	13.7 ± 0.4
100	1.23 ± 0.19	12.6 ± 0.9
200	1.10 ± 0.15	7.3 ± 0.4

1 *K*_M_ and *V*_MAX_ values of the chromogenic substrate hydrolysis by CatG were measured as described under “Methods” section. mAU indicates milliabsorbance units.

2 Error represents ± 1 S.E.

**Table 3:** Direct inhibition of CatG by SPGG under salt concentrations^1^

Molecule	[NaCl] (mM)	IC_50_ (nM)	HS	Δ*Y* (%)
SPGG	50	42 ± 8^2^	0.7 ± 0.1	102 ± 5
	100	57 ± 5	1.1 ± 0.1	90 ± 2
	200	76 ± 7	1.6 ± 0.2	83 ± 2
	400	130 ± 20	1.2 ± 0.2	92 ± 4

1 The inhibition parameters were obtained following non-linear regression analysis of direct inhibition of CatG. Enzyme inhibition was evaluated by spectrophotometric analysis of residual CatG activity.

2 Errors represent ± 1 S.E.

**Table 4:** Salt-dependent calculated affinities of SPGG at pH 7.4 and 37°C^1^

[NaCl] (mM)	*IC_50_* (nM)	Calculated *K_i_* (nM)	Slope^2^	*Z* ^2^	Intercept^2^	*K*_*i*(non-ionic)_ (μM)	Δ*G*_(non-ionic)_ (kcal/mol)	*ΔG*_(non-ionic)_ (%)^3^
50	42 ± 8^4^	32 ± 6	0.621	0.77625	−6.7034	0.19797	9.498019	91.5
100	57 ± 5	47 ± 4						
200	76 ± 7	66 ± 6						
400	130 ± 20	120 ± 18						

1 The *K*_i_ (nM) values were estimated using the equation in https://bioinfo-abcc.ncifcrf.gov/IC50_Ki_Converter/index.php.^[18]^.

2 Slope, *Z* and intercept were calculated from linear regressional analysis of log *K_i_* calculated versus log[NaCl] as defined by equation: Log (*K_i_* (M)) = Log (*K_i_* (M) (non-ionic)) + *Zψ* Log ([NaCl] (M)); *ψ* = 0.8.^[19]^

3 Nonionic binding energy contribution to the total is expressed as percentage.

4 Error represent standard error calculated using global fit of the data.

## Data Availability

Data related to this study is included in the article.
