# Supplementary material for: Sulphated penta-galloyl glucopyranoside (SPGG) is glycosaminoglycan mimetic allosteric inhibitor of cathepsin G
Source: RPS Pharm Pharmacol Rep. Author manuscript; Available in PMC 2023 Feb 22. (PMC9942669; doi:10.1093/rpsppr/rqad001)
Supplement: Suppl Info [file NIHMS1875365-supplement-Suppl_Info.docx]

**Sulfated Penta-Galloyl Glucopyranoside (SPGG) Is Glycosaminoglycan Mimetic**

**Allosteric Inhibitor of Cathepsin G**

Rami A. Al-Horani,^1#^ Daniel K Afosah,^2^ Srabani Kar,^1^ Kholoud F. Aliter,^3^ Madhusoodanan Mottamal^4^

*^1^Division of Basic Pharmaceutical Sciences, College of Pharmacy, Xavier University of Louisiana, New Orleans LA 70125*

*^2^Department of Medicinal Chemistry, School of Pharmacy, Virginia Commonwealth University, Richmond VA 23219*

*^3^Department of Chemistry, School of STEM, Dillard University, New Orleans LA 70122*

*^4^Department of Chemistry, Xavier University of Louisiana, New Orleans LA 70125*

**Table S1. Enzymes and Chromogenic substrates.**

| **Enzyme** | **Enzyme Conc.** | **Substrate** | **Substrate Conc.** | **Formula** |
| --- | --- | --- | --- | --- |
| CatG | 30 nM | S-7388 | 750 µM | N-Succinyl-L-alanyl-L-alanyl-L-prolyl-L-phenylalanine 4-nitroanilide |
| Thrombin | 6 nM | Spectrozyme TH | 50 µM | H-D-cyclohexylalanyl-alanyl-argininepara-nitroanilide diacetate salt |
| FVIIa | 8 nM | Spectrozyme FVIIa | 1000 µM | Methanesulphonyl-D-cyclohexyl-alanyl-butyl-arginine-para-nitroaniline monoacetate salt |
| FIXa | 89 nM | Spectrozyme FIXa | 850 µM | Methylsulfonyl-D-cyclohexyl glycyl-glycyl-arginine-para-nitroanilide monoacetate salt |
| FXa | 1.09 nM | Spectrozyme FXa | 125 µM | Methoxycarbonyl-D-cyclohexylglycylglycyl-arginine-para-nitroanilide acetate |
| FXIa | 0.765 nM | S-2366 | 345 µM | L-Pyroglutamyl-L-prolyl-L-argininep-Nitroaniline hydrochloride |
| FXIIa | 5 nM | Spectrozyme FXIIa | 125 µM | H-D-CHA-Gly-Arg-pNA 2AcOH |
| Trypsin | 3 nM | S-2222 | 80 µM | N-Benzoyl-L-isoleucyl-L-glutamylglycyl-L-arginine-p-nitroaniline hydrochloride and its methyl ester |
| Chymotrypsin | 20 nM | Spectrozyme CTY | 240 µM | HBR H-Gly-Pro-Phe-pNA |
